# Notice upon Some of the Diseases of the Mouth, &c.

**Published:** 1849-07

**Authors:** 


*Notice upon Some of the Diseases of the Mouth, &c. &c.* By HATTUTE, Surgeon Dentist to the State Major General of the First Military Division, residing in Galirie, pp. 32, 8vo. Paris, 1847.

The above is the title of a pamphlet which we have recently received from Paris. It is designed for the general reader, and treats in a very brief manner on a few of the diseases of the teeth and gums, as also upon artificial teeth. From the hasty perusal which we have given it, we are inclined to believe that it is intended more for an advertisement, than to impart useful and correct inflammation.—*Balt. Ed.*

